# Retraction: *Wolbachia* Transcription Elongation Factor “Wol GreA” Interacts with α2ββ′σ Subunits of RNA Polymerase through Its Dimeric C-Terminal Domain

**DOI:** 10.1371/journal.pntd.0010694

**Published:** 2022-08-08

**Authors:** The PLOS Neglected Tropical Diseases Editors

Following the publication of this article [1], concerns were raised regarding results presented in Figs 1, 6 and 8. Specifically,

- In Fig 1D, when colour levels are adjusted to visualise the background, there is an irregular rectangular patch of background noise in lanes 2 and 3 that does not appear to match the overall background noise of the figure.
- In Fig 6B, when colour levels are adjusted to visualise the background, the noise in the background directly surrounding the bands in the Marker lane (M) and the background directly surrounding the bands detected around the 10kDa marker (lanes 1–8) do not appear to match the overall background noise of the figure.
- In Fig 6C, when colour levels are adjusted to visualise the background, the noise in the background of the second lane (1) does not appear to match the overall background noise of the figure. In addition, the kDa values assigned to the marker lanes appear to be misaligned.
- In Fig 8C, the results presented in the lanes marked with 1, 2, and 3 appear similar, even though these results represent independent samples.
- In Fig 8D, the results presented in the lanes marked as 2 and 3 appear similar, even though these results represent independent samples.

The first author indicated that the original data underlying the results presented in Figs 1D, 8B, 8C and 8D are no longer available, and provided repeat experiment data instead. The underlying data provided for Fig 6B did not resolve the concerns, and the underlying data provided for Fig 6C confirm that the image manipulations for this figure are not in line with the journal’s image manipulation guidelines. In the absence of the original data underlying Figs 1D, 8C, and 8D, the concerns with these figures cannot be resolved.

In light of the concerns affecting multiple figure panels that question the reliability of these data, the *PLOS Neglected Tropical Diseases* Editors retract this article.

SMB agreed with the retraction. NS, DC, CLG, and PB either did not respond directly or could not be reached. JKN did not agree with the retraction.
